# Discordant p53 and BRG1 expression in synchronous low-grade uterine endometrioid carcinoma and SMARCA4-deficient ovarian undifferentiated carcinoma: a case report

**DOI:** 10.1186/s12905-026-04415-0

**Published:** 2026-03-29

**Authors:** Yuchen Sun, Jianyi Sun, Yuning Xie, Yifei Wang, Rui Ma, Zhiyong Liu, Li Xu, Shujuan Yao, Minmin Yu, Wei Shi

**Affiliations:** 1 Affiliated Hospital of Shandong University of Traditional Chinese Medicine, Jinan, Shandong 250014 China; 2 Shandong University of Traditional Chinese Medicine, Jinan, Shandong 250355 China; 3 Shandong Provincial Maternity and Child Health Care Hospital, Jinan, Shandong 250013 China; 4 Shandong Provincial Hospital of Integrated Traditional Chinese and Western Medicine, Jinan, Shandong 250001 China

**Keywords:** Synchronous multiple primary malignancies, Endometrioid carcinoma, SMARCA4-deficient undifferentiated carcinoma, Loss of BRG1 protein expression, p53-mutant phenotype, Mismatch repair status

## Abstract

**Background:**

Synchronous multiple primary malignancies (SMPMs) involving the uterus and ovary pose significant diagnostic challenges, particularly in distinguishing independent primary tumors from metastatic disease when tumors exhibit overlapping or divergent morphologic and molecular features. Accurate assessment of tumor clonality increasingly relies on integrated clinicopathologic and molecular profiling, which has important implications for staging, prognosis, and therapeutic decision-making.

**Case presentation:**

We describe a 47-year-old woman presenting with abdominal distension and extensive peritoneal dissemination. Imaging revealed a uterine endometrial mass and bilateral adnexal lesions. Following neoadjuvant chemotherapy, she underwent optimal cytoreductive surgery. Histopathologic examination demonstrated a FIGO stage IA low-grade endometrioid carcinoma of the uterine corpus and a SMARCA4-deficient undifferentiated carcinoma of the ovary. Immunohistochemistry showed that the uterine tumor exhibited wild-type p53 expression, retained BRG1 expression, hormone receptor positivity, and proficient mismatch repair (pMMR) status. In contrast, the ovarian tumor demonstrated a p53-mutant phenotype with diffuse strong nuclear staining, complete loss of BRG1 expression, and pMMR status. Multiple foci of endometriosis were also identified in the pelvis.

**Conclusions:**

The marked molecular heterogeneity observed in this case, particularly the discordant p53 and BRG1 expression patterns, supports the interpretation of two independent primary malignancies rather than metastatic spread from a common origin. This case highlights the value of integrated clinicopathologic and immunohistochemical assessment, including evaluation of p53, BRG1, and MMR status, in the diagnostic work-up of synchronous gynecologic tumors. Such an approach may contribute to more accurate diagnosis, risk stratification, and individualized treatment planning.

**Supplementary Information:**

The online version contains supplementary material available at 10.1186/s12905-026-04415-0.

## Background

SMPMs are defined as two or more distinct primary tumors occurring in the same individual, either simultaneously or within a short interval (usually 6 months) [1–3]. While relatively uncommon, their incidence has been increasing, partly due to improved diagnostic techniques and increased life expectancy [4, 5]. In the gynecological field, synchronous endometrial and ovarian carcinomas represent the most common type of SMPMs, accounting for 5–10% of all endometrial cancers and up to 10% of endometrioid ovarian cancers [6–8]. These synchronous tumors often pose a diagnostic challenge in distinguishing between independent primary tumors and metastatic disease, with significant implications for prognosis and treatment planning [9–11].

Traditionally, histological similarities, tumor grade, and depth of myometrial invasion have been used to differentiate between these entities. However, with advances in molecular pathology, an increasing number of studies emphasize the critical role of molecular profiling in elucidating tumor clonality and biological behavior [12–14]. We present a case of synchronous low-grade endometrioid carcinoma of the uterine corpus and SMARCA4-deficient undifferentiated carcinoma of the ovary. This case is remarkable for the extensive disease presentation at diagnosis, the simultaneous occurrence of histologically disparate tumors, and, most significantly, their strikingly discordant molecular profiles, most notably concerning p53 immunohistochemical (IHC) expression and the status of SWI/SNF chromatin remodeling complex components (BRG1), alongside mismatch repair (MMR) status [15–17]. The differential expression of these markers supports the interpretation of independent primary tumors and contributes to accurate diagnosis and individualized treatment planning. Additionally, the presence of concurrent endometriosis further enriches the clinical context of this uncommon presentation. BRG1 has been implicated in endometrial tumor biology [18]. To our knowledge, few reports have specifically highlighted SMARCA4-deficient undifferentiated carcinoma occurring synchronously with uterine endometrioid carcinoma, underscoring the potential diagnostic value of this marker.

## Methods

Immunohistochemical staining was performed on formalin-fixed, paraffin-embedded tissue sections using standard diagnostic protocols. The primary antibodies included ER, PR, p53, BRG1 (SMARCA4), INI1 (SMARCB1), MLH1, PMS2, MSH2, MSH6, Ki-67, P16, WT1, PAX8, HNF1-β, Napsin-A, CK, CgA, Syn, CD56, and Vimentin. Detailed information regarding antibody sources, clone numbers, dilutions, and detection systems is provided in Supplementary Table 1. Immunostaining was performed using an automated staining platform with a polymer-based detection system according to the manufacturer’s instructions.

Staining results were independently evaluated by experienced pathologists according to established diagnostic criteria. Nuclear staining was considered positive for BRG1, INI1, and mismatch repair (MMR) proteins when present in tumor cells with appropriate internal controls. Loss of expression was defined as complete absence of nuclear staining in tumor cells with retained staining in internal control cells. The p53 staining pattern was categorized as wild-type, overexpression (diffuse strong nuclear staining), or complete absence (null pattern) according to accepted interpretation guidelines. The Ki-67 labeling index was recorded as the percentage of positively stained tumor nuclei.

## Case presentation

**Fig. 1:**
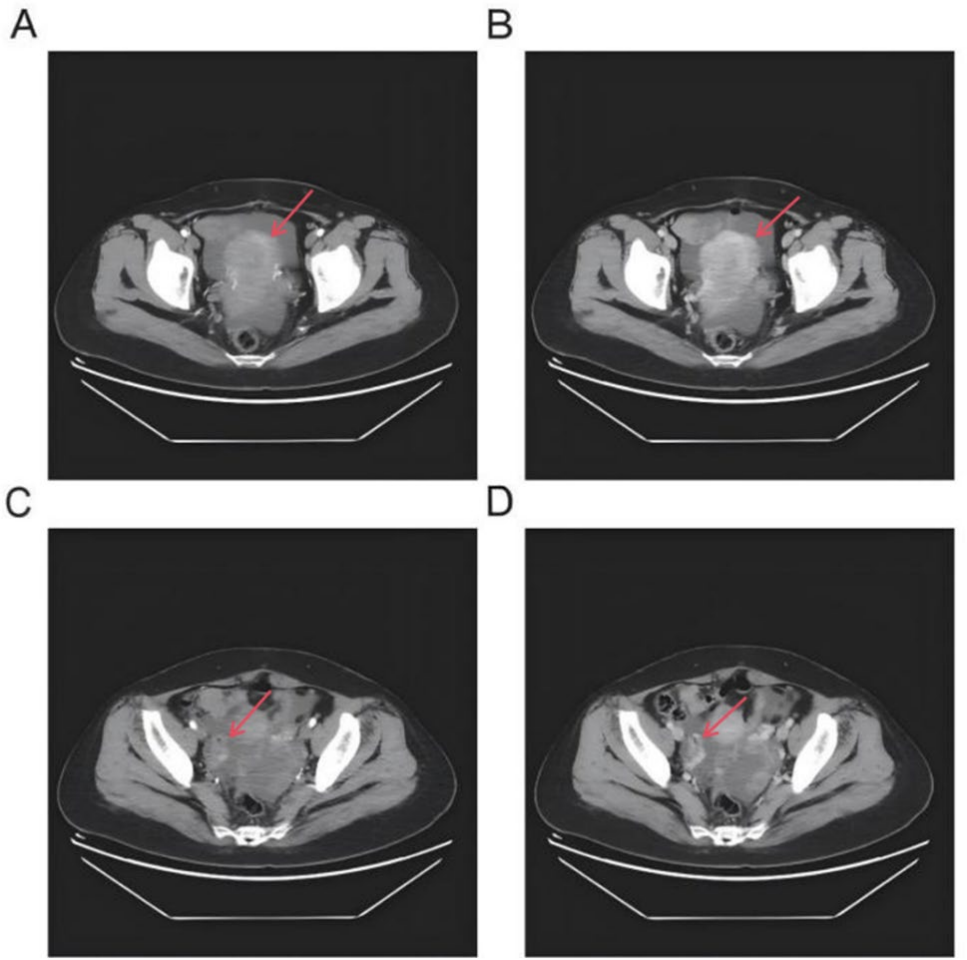
Representative pre-operative abdominopelvic computed tomography (CT) images showing the uterine lesion (**A**-**B**) and the ovarian mass (**C**-**D**). Arrows indicate the primary tumors. The red arrows highlight the specific locations of the tumors in both the uterine and ovarian regions

A 47-year-old woman presented to our hospital with a diagnosis of endometrial carcinoma and an adnexal mass. Her medical history was unremarkable. At initial presentation, an endometrial biopsy demonstrated endometrioid adenocarcinoma. Concurrent imaging studies, including an abdominopelvic CT scan, revealed a highly suspicious solid-cystic mass in the left adnexa measuring approximately 8.8 × 8.0 × 6.4 cm, along with prominent heterogeneous enhancement within the uterine endometrium suggestive of an endometrial lesion (Fig. 1). In addition, extensive peritoneal thickening and nodularity (largest lesion 5.9 cm in the mesentery), ascites, and a suspicious right upper-lobe lung nodule were noted. Subsequent gynecological ultrasound confirmed a left adnexal mass highly suspicious for malignancy, characterized by multiple cystic areas, along with heterogeneous endometrial echoes and pelvic free fluid. Preoperative serum tumor markers, including CA-125, were not available in this case. Preoperative cardiac evaluation, including electrocardiography, echocardiography, and lower-extremity venous ultrasound, was largely unremarkable except for mild valvular regurgitation, and the patient was considered fit for surgery.

Given the advanced stage at presentation, the patient received four cycles of neoadjuvant chemotherapy over a period of approximately 3 months, starting in January 2025, followed by extensive cytoreductive surgery on April 15, 2025. After surgery, she began adjuvant chemotherapy with paclitaxel and carboplatin, which was modified to include bevacizumab for the final two cycles. At the time of reporting, she was approximately 3 months post-surgery (i.e., in July 2025) and had completed three cycles of postoperative therapy.

### Uterine corpus

Histopathological examination of the uterine corpus revealed a low-grade endometrioid carcinoma with mucinous differentiation and focal squamous metaplasia, invading the superficial myometrium (less than half of the myometrial thickness). Hematoxylin and eosin staining showed well-differentiated glandular architecture with mild nuclear atypia. Based on the superficial myometrial invasion, the uterine tumor was consistent with FIGO stage IA.

**Fig. 2:**
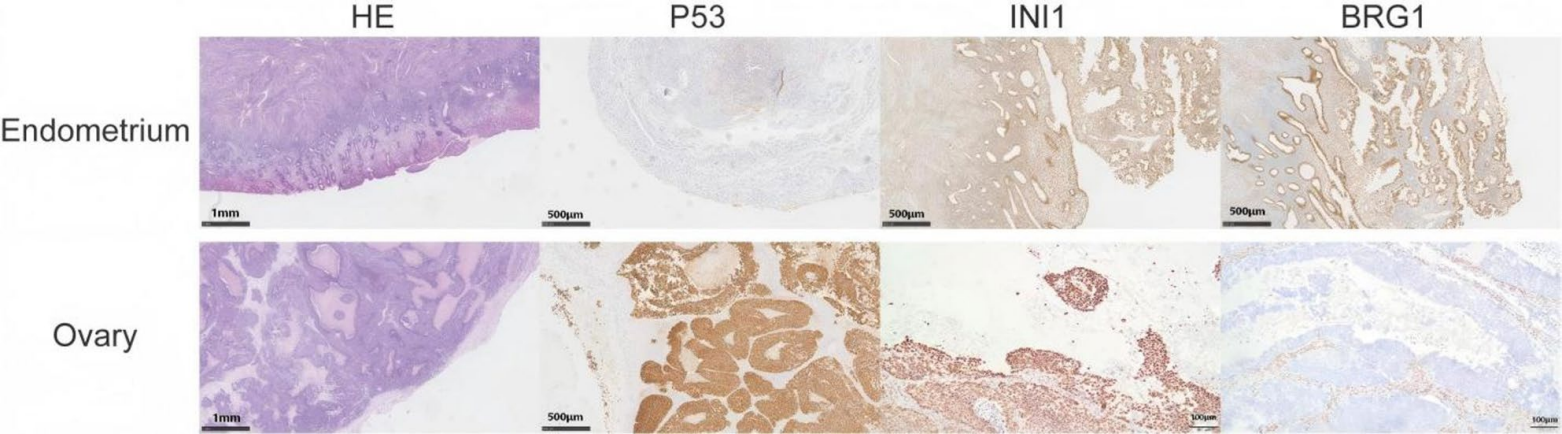
Histopathological and immunohistochemical findings of uterine and ovarian tumors. Scale bars: 1 mm for HE images, 500 μm and 100 μm for immunohistochemistry images

Immunohistochemistry demonstrated partial positivity for ER and PR, patchy p16 expression, and a Ki-67 proliferation index of approximately 60%. All mismatch repair (MMR) proteins (MLH1, PMS2, MSH2, and MSH6) showed intact nuclear expression in tumor cells with appropriate internal controls, consistent with proficient mismatch repair (pMMR) status. BRG1 and INI1 expression were retained in tumor nuclei. These findings supported the diagnosis of low-grade endometrioid carcinoma of the uterus (Fig. 2).

### Ovary

Histopathological examination of the left ovary demonstrated a poorly differentiated malignant neoplasm with predominantly solid growth and marked nuclear pleomorphism. Extensive tumor involvement was identified in the omentum, left paracolic region of the sigmoid colon, anterior rectal wall, and diaphragm, consistent with metastatic spread. Immunohistochemical analysis showed positivity for cytokeratin (CK) and negativity for ER, PR, WT1, HNF1-β, PAX8, CgA, Synaptophysin, CD56, Vimentin, and Napsin-A. The tumor exhibited patchy p16 expression and a high Ki-67 labeling index of approximately 70%. p53 immunostaining revealed a diffuse strong nuclear staining pattern consistent with a p53-mutant phenotype. Notably, BRG1 (SMARCA4) expression was completely lost in tumor nuclei, while INI1 expression was retained. All MMR proteins (MLH1, PMS2, MSH2, and MSH6) showed intact nuclear expression, indicating a proficient mismatch repair (pMMR) status. Although the tumor demonstrated diffuse p53 overexpression, the absence of WT1 expression, non-diffuse p16 staining, and complete loss of BRG1 expression did not support a diagnosis of conventional high-grade serous carcinoma. Instead, the overall morphologic and immunophenotypic features were consistent with SMARCA4-deficient undifferentiated carcinoma.

### Left fallopian tube

The left fallopian tube showed focal epithelial atypia with wild-type p53 expression.

### Metastatic lesions

Metastatic carcinoma was identified in the diaphragm, anterior rectal wall, left sigmoid colon, and omentum. These lesions demonstrated morphologic features consistent with the ovarian carcinoma and were distinct from the uterine tumor, supporting attribution to the ovarian primary.

### Endometriosis

Foci of endometriosis were identified in the bladder reflection peritoneum, left bladder surface peritoneum, and the posterior leaf of the right broad ligament.

The cervical outer os, right fallopian tube, and right ovary were negative for malignancy.

### Final diagnosis

SMARCA4-deficient undifferentiated carcinoma of the left ovary (FIGO stage IIIC) with metastatic involvement, and low-grade endometrioid carcinoma of the uterus (FIGO stage IA), status post neoadjuvant chemotherapy and cytoreductive surgery.

## Discussion

This case represents a diagnostically challenging scenario of synchronous low-grade endometrioid carcinoma of the uterine corpus and SMARCA4-deficient undifferentiated carcinoma of the ovary with extensive peritoneal dissemination at presentation. The coexistence of two histologically distinct tumors prompted careful evaluation of whether they represented independent primaries or metastatic disease.

Molecular Heterogeneity in Synchronous Gynecologic Tumors.

The coexistence of synchronous uterine and ovarian malignancies often necessitates careful distinction between independent primary tumors and metastatic spread [19, 20]. Historically, criteria such as differing histologic types, discordant tumor grades, and limited myometrial invasion have supported the interpretation of independent primaries [19, 21, 22]. In the present case, the tumors were histologically distinct, consisting of a low-grade endometrioid carcinoma of the uterus and a SMARCA4-deficient undifferentiated carcinoma of the ovary.Although both tumors showed proficient mismatch repair status, their p53 and BRG1 expression patterns differed substantially. The uterine carcinoma exhibited a wild-type p53 pattern with retained BRG1, whereas the ovarian carcinoma showed mutation-type p53 expression and loss of BRG1.

### p53 status and biological implications

p53 plays a central role in maintaining genomic stability, and its immunohistochemical expression pattern serves as a surrogate marker for TP53 mutational status [23, 24]. In this case, the ovarian SMARCA4-deficient carcinoma demonstrated diffuse strong nuclear staining consistent with a p53-mutant phenotype, which is frequently associated with aggressive biological behavior and genomic instability [25, 26]. In contrast, the uterine low-grade endometrioid carcinoma showed a wild-type p53 pattern, typically seen in hormonally driven, lower-grade endometrial tumors.

### SWI/SNF complex deficiency in the ovarian carcinoma

The most distinctive molecular feature in this case was the complete loss of BRG1 (SMARCA4) expression in the ovarian tumor, while BRG1 expression was retained in the uterine carcinoma. The SWI/SNF chromatin remodeling complex, which includes BRG1 (SMARCA4) and INI1 (SMARCB1), plays a critical role in transcriptional regulation and tumor suppression [27, 28]. Loss of SMARCA4 function has been described in a subset of highly aggressive undifferentiated and rhabdoid-like malignancies, including ovarian tumors [29, 30]. In the present case, the combination of diffuse p53 overexpression, BRG1 loss, ER/PR negativity, WT1 negativity, and lack of typical serous morphology supported a diagnosis of SMARCA4-deficient undifferentiated carcinoma rather than high-grade serous or endometrioid carcinoma.

### Differential diagnosis

Diffuse p53 overexpression in the ovarian tumor raises the differential diagnosis of high-grade serous carcinoma. However, the lack of WT1 expression, non-diffuse p16 staining, absence of classic serous morphology, and complete loss of BRG1 expression argue against conventional high-grade serous carcinoma. Instead, the overall morphologic and immunophenotypic features are more consistent with SMARCA4-deficient undifferentiated carcinoma.

### Association with endometriosis

The concomitant presence of multiple peritoneal foci of endometriosis in this patient is noteworthy. Endometriosis, particularly ovarian endometriosis, is a well-established precursor lesion for certain ovarian carcinomas, most commonly endometrioid and clear cell subtypes [31–33].In the present case, however, the ovarian tumor was diagnosed as SMARCA4-deficient undifferentiated carcinoma rather than a conventional endometrioid carcinoma. Therefore, a direct etiologic relationship between the observed endometriotic foci and the ovarian malignancy cannot be established.Nonetheless, the coexistence of endometriosis and ovarian carcinoma may reflect a complex pelvic microenvironment characterized by chronic inflammation and altered molecular signaling pathways [34, 35]. Further investigation would be required to clarify whether endometriosis plays any contributory role in the pathogenesis of SMARCA4-deficient ovarian tumors.

### Treatment implications and future directions

The patient presented with advanced disease requiring multimodal treatment, including neoadjuvant chemotherapy, cytoreductive surgery, and adjuvant chemotherapy with bevacizumab. Both tumors demonstrated proficient mismatch repair status; therefore, immune checkpoint inhibition would not be routinely indicated based solely on current findings.The ovarian carcinoma exhibited p53 mutation-type expression and SMARCA4 deficiency, both of which are associated with aggressive tumor biology. Although targeted therapies directly addressing TP53 alterations or SWI/SNF complex deficiency remain under investigation, these molecular characteristics may become clinically relevant as precision oncology evolves.

### Limitations

A major limitation of this report is that tumor relatedness was inferred primarily from morphologic and immunohistochemical findings, without the benefit of PCR-based microsatellite instability testing or next-generation sequencing. Given the lack of confirmatory genomic analyses, the interpretation of independent primary tumors should be regarded as supportive rather than definitive. Additional genomic studies would be necessary to more conclusively establish the clonal relationship between the two neoplasms and strengthen clonality assessment in similar cases.

## Conclusion

We report a case of synchronous low-grade endometrioid carcinoma of the uterus and SMARCA4-deficient undifferentiated carcinoma of the ovary exhibiting distinct immunophenotypic profiles, including divergent p53 patterns and differential BRG1 expression. These findings support the interpretation of biologically independent tumors, but this conclusion should be considered tentative in the absence of confirmatory genomic testing. This case highlights the potential diagnostic value of incorporating SWI/SNF component analysis in selected synchronous gynecologic neoplasms, but further studies with genomic validation are necessary to conclusively establish the clonal relationship.

## Supplementary Information

Supplementary Material 1.

## Data Availability

The original contributions presented in the study are included in the article. Further inquiries can be directed to the corresponding author.

## Abbreviations

BRG1: Brahma-related gene 1
ER: Estrogen receptor
FIGO: International Federation of Gynecology and Obstetrics
INI1: Integrase interactor 1
MMR: Mismatch repair
pMMR: proficient mismatch repair
PR: Progesterone receptor
SMARCA4: SWI/SNF-related matrix-associated actin-dependent regulator of chromatin subfamily A member 4
SMPMs: Synchronous multiple primary malignancies
SWI/SNF: SWItch/Sucrose Non-Fermentable chromatin-remodeling complex
