# Supplementary material for: Discordant p53 and BRG1 expression in synchronous low-grade uterine endometrioid carcinoma and SMARCA4-deficient ovarian undifferentiated carcinoma: a case report
Source: BMC Womens Health. 2026 Mar 29;26:237. doi: 10.1186/s12905-026-04415-0 (PMC13154530; doi:10.1186/s12905-026-04415-0)
Supplement: Supplementary file 1 — Supplementary Material 1. [file 12905_2026_4415_MOESM1_ESM.doc]

| **Marker** | **Supplier** | **Catalog ##** | **Clone** | **Host** | **Dilution** | **Notes** |
| --- | --- | --- | --- | --- | --- | --- |
| ER | Proteintech | **15550-1-AP** | Polyclonal | Rabbit | 1:100 | IHC validated |
| PR | Proteintech | **55260-1-AP** | Polyclonal | Rabbit | 1:100 | IHC validated |
| p53 | Proteintech | **60283-2-Ig** | **6C4B6** | Mouse | 1:200 | IHC validated |
| BRG1/SMARCA4 | Proteintech | **17454-1-AP** | Polyclonal | Rabbit | 1:200 | IHC |
| INI1/SMARCB1 | Proteintech | **60361-1-Ig** | Polyclonal | Mouse | 1:200 | IHC |
| MLH1 | Proteintech | **13531-1-AP** | Polyclonal | Rabbit | 1:100 | IHC |
| PMS2 | Proteintech | **14610-1-AP** | Polyclonal | Rabbit | 1:100 | IHC |
| MSH2 | Proteintech | **11792-1-AP** | Polyclonal | Rabbit | 1:100 | IHC |
| MSH6 | Proteintech | **10797-1-AP** | Polyclonal | Rabbit | 1:100 | IHC |
| Ki-67 | Proteintech | **32270-1-AP** | Polyclonal | Rabbit | 1:500 | IHC |
| P16 | Proteintech | **10883-1-AP** | Polyclonal | Rabbit | 1:100 | IHC |
| WT1 | Proteintech | **22666-1-AP** | Polyclonal | Rabbit | 1:100 | IHC |
| PAX8 | Proteintech | **15998-1-AP** | Polyclonal | Rabbit | 1:100 | IHC |
| HNF1-β | Proteintech | **15048-1-AP** | Polyclonal | Rabbit | 1:100 | IHC |
| Napsin-A | Proteintech | **21740-1-AP** | Polyclonal | Rabbit | 1:200 | IHC |
| CK(Pan-Cytokeratin) | Proteintech | **66031-1-Ig** | Polyclonal | Mouse | 1:200 | IHC |
| CgA(Chromogranin A) | Proteintech | **11244-1-AP** | Polyclonal | Rabbit | 1:100 | IHC |
| Syn(Synaptophysin) | Proteintech | **66493-1-Ig** | Polyclonal | Mouse | 1:100 | IHC |
| CD56 | Proteintech | **22034-1-AP** | Polyclonal | Rabbit | 1:100 | IHC |
| Vimentin | Proteintech | **10366-1-AP** | Polyclonal | Rabbit | 1:200 | IHC |

Supplementary Table 1
